# Premenopausal women with pelvic organ prolapse and pelvic floor symptoms at a tertiary hospital in Thailand: a retrospective cohort study

**DOI:** 10.1080/07853890.2025.2561796

**Published:** 2025-09-30

**Authors:** Apisith Saraluck, Jittima Manonai, Komkrit Aimjirakul, Rujira Wattanayingcharoenchai, Orawee Chinthakanan

**Affiliations:** Division of Female Pelvic Medicine and Reconstructive Surgery, Department of Obstetrics and Gynaecology, Faculty of Medicine Ramathibodi Hospital, Mahidol University, Bangkok, Thailand

**Keywords:** Pelvic organ prolapse, pelvic floor symptoms, premenopausal

## Abstract

**Aim:**

Pelvic organ prolapse (POP) primarily affects older women, with limited data on premenopausal cases, especially among Asians. Severe pelvic floor symptoms, including urinary, bowel, and sexual dysfunction, remain understudied in this population. This study aimed to describe symptom severity and treatment outcomes in premenopausal POP patients.

**Methods:**

A retrospective cohort study was conducted on premenopausal POP patients at a university hospital over six years. The Pelvic Floor Bother Questionnaire (PFBQ) assessed symptom severity, and treatment outcomes were evaluated over two years. Data were presented as counts with percentages or means with standard deviations.

**Results:**

Among 87 women (mean age 44.8 ± 4.9 years), 12 (13.8%) had stage I, 47 (54.0%) stage II, 24 (27.7%) stage III, and 4 (4.6%) stage IV POP. Common comorbidities included dyslipidemia (13.8%), diabetes mellitus (11.5%), and thyroid disease (10.3%). Six patients had connective tissue diseases or nerve damage. Premenopausal women accounted for approximately 5.6% (87 out of 1,567) of all new POP cases in this population. The most bothersome symptoms were prolapse bulging (83.9%), urinary frequency (58.6%), and urgency (49.4%). Treatment included pelvic floor muscle training (10%), pessary use (28%), and surgery (62%). Vaginal hysterectomy with pelvic floor repair was most common, with 8 colpocleisis cases. Symptom improvement at two years was highest after surgery (90.1%), followed by pessary (82.6%) and pelvic floor training (68.3%).

**Conclusion:**

This study highlights the burden of POP and pelvic floor symptoms in Thai women, emphasizing the need for tailored management strategies.

## Introduction

Pelvic organ prolapse (POP) is a gynecological disorder characterized by the herniation of pelvic organs into the vagina as a result of weakened ligaments or muscles [1]. The classification of POP is based on the specific compartment of origin, which includes anterior compartment prolapse, posterior compartment prolapse, and apical compartment prolapse. Women with POP may present a variety of intense pelvic floor symptoms, such as urinary, bowel, or sexual dysfunction, leading to a reduced quality of life and withdrawal from social activities. A mix of anatomical, physiological, genetic, behavioral, and reproductive factors interacts during a woman’s lifetime to likely cause pelvic floor dysfunction [2]. Prior research has demonstrated that risk factors associated with POP encompass characteristics such as the number of pregnancies, vaginal childbirth, advanced age, body mass index (BMI), engaging in physically demanding jobs on a regular basis, and inadequate nutrition [3]. These factors further contribute to the development of POP. The reported prevalence of POP varies widely depending on the method of assessment. A recent international consultation reported that prevalence estimates range from 1 to 65%, with symptom-based surveys yielding rates between 1–31%, physical examination-based studies between 10–50%, and combined assessments between 20–65%. Most existing population-based studies rely on self-reported symptoms and do not include objective examination data [4]. Although population-based data remain limited, several studies from developing countries have reported a substantial proportion of premenopausal women among those presenting with advanced POP, particularly in surgical cohorts. For example, a prospective study in the Democratic Republic of the Congo found that 25% of women undergoing POP surgery were premenopausal [5], and a study from Ethiopia also reported advanced POP in premenopausal women [6]. The impact of POP on younger women has been increasingly recognised, with studies showing that prolapse symptoms can significantly affect physical function, body image, and quality of life [7,8]. In Thailand, institutional data from a university hospital cohort have reported on pelvic floor symptoms associated with POP [9,10], although national population-based estimates are lacking. Furthermore, the intensity of pelvic floor symptoms and the adverse impact on sexual function in premenopausal women with POP have been underrecognized and underreported in existing literature. The impact on POP and pelvic floor symptoms may vary between premenopausal and postmenopausal women, particularly in terms of concerns, counseling responsibilities, and management choices. The cultural context of the region and country also influences this variation, in addition to age. Within Thailand, the majority of patients diagnosed with POP are said to be postmenopausal women, according to the research. The incidence of premenopausal women with POP is estimated less than 5%. Nevertheless, there was a lack of information regarding the specific kind and extent of POP in premenopausal women. Furthermore, the examination of the extent of seriousness in each compartment and the consequences on the spectrum of severe pelvic floor symptoms, such as urine symptoms, difficulties with bowel movements, and sexual function, has been restricted among the Asian population. In Thailand, there are currently no national clinical guidelines specific to the diagnosis and management of POP. At our institution, clinical care is based on international standards, particularly those recommended by the International Urogynecological Association (IUGA), including use of the POP-Q system for prolapse staging [1]. Treatment selection follows a shared decision-making model involving physicians, patients, and family members. All available options, ranging from pelvic floor muscle training and pessary use to surgery, are explained in detail, including potential benefits, limitations, and long-term outcomes. This patient-centered approach ensures individualized treatment aligned with personal needs and clinical presentation. This study aims to assess the extent of pelvic organ prolapse, pelvic floor symptoms, and the effectiveness of different treatment options in premenopausal women diagnosed with POP. This material will be advantageous for the comprehensive care in counseling and management of premenopausal women with POP.

## Materials and methods

### Study design and setting

This was a retrospective cohort study conducted at the Urogynecology Clinic, Ramathibodi Hospital, Mahidol University, a university-affiliated tertiary referral center in Bangkok, Thailand. The study covered patient records from January 2016 to December 2020.

### Ethical approval

This study was approved by the Ethical Clearance Committee on Human Rights Related to Research Involving Human Subjects, Faculty of Medicine, Ramathibodi Hospital, Mahidol University (Approval No. MURA2024/30). All patient data were anonymized prior to analysis.

### Participants

Eligible participants were premenopausal women aged between 18 and 55 years, diagnosed with POP in any compartment, anterior, posterior, or apical, based on clinical examination, and who had complete follow-up data at 3, 12, and 24 months post-treatment. These timepoints reflect the standard care protocol at our urogynecology clinic, which includes proactive appointment scheduling, reminder calls or mobile notifications, and systematic documentation in the electronic medical record system. The stage and compartment of pelvic organ prolapse were assessed using the Pelvic Organ Prolapse Quantification (POP-Q) system [11] during standardized pelvic examinations, performed by urogynecologists (University Lecturer). Menopausal status was defined as the complete absence of menstruation for at least 12 consecutive months [12]. Women exhibiting perimenopausal features, such as irregular cycles or vasomotor symptoms, were excluded. Exclusion criteria included incomplete clinical data.

### Data collection

Data were obtained from two sources: (1) paper-based intake forms completed during clinic visits, and (2) electronic medical records within the hospital’s data management system. Extracted variables included age, parity, menopausal status, medical and surgical history (including prior vaginal delivery and hysterectomy), physical examination findings, and details of POP treatment.

### Symptom assessment

Pelvic floor symptoms and their impact were assessed using the Thai version of the Pelvic Floor Bother Questionnaire (PFBQ). The original English-language PFBQ, developed at the Cleveland Clinic, consists of nine self-administered items evaluating prolapse, bladder, and bowel symptoms [13]. The Thai version was developed through forward and back translation and validated by three urogynecology experts. Its internal consistency was previously confirmed (reliability coefficient = 0.95) [9].

### Statistical analysis

Data analysis was performed using STATA version 16.0 (StataCorp LLC, College Station, TX, USA). Descriptive statistics were applied. Categorical variables are presented as frequencies and percentages, while continuous variables are expressed as means with standard deviations. For the analysis of baseline pelvic floor symptoms, all 87 women were included regardless of subsequent treatment changes (intention-to-treat). For the analysis of symptom improvement across follow-up, results were reported using an as-treated approach, with percentages calculated only among women who continued the same treatment modality at each time point. Women who discontinued or switched treatment were included in baseline analyses according to their initial treatment group, but were not counted in subsequent improvement rate calculations after changing modality. This approach was chosen to ensure clarity and consistency in reporting outcomes.

## Results

From January 2016 to December 2020, a tertiary hospital in Bangkok, Thailand, Ramathibodi Hospital, Mahidol University, diagnosed 87 premenopausal women with pelvic organ prolapse, during which a total of 1,567 new POP cases were recorded. In this study, the premenopausal women had a mean age of 44.81 ± 4.90 years and a mean body mass index of 24.48 ± 3.28 kg/m^2^. Regarding parity, 8.0% of participants were nulliparous, while 18.4% were primiparous. The majority (73.6%) were multiparous, with most having had two births (62.1%). A smaller proportion had three (6.9%), four (3.4%), or five births (1.1%), and 79 (90.80%) had previous vaginal delivery. Regarding the underlying conditions related to the study participants, the most commonly reported conditions were dyslipidemia (13.8%), diabetes mellitus (11.5%), and thyroid disease (10.3%). Two patients (2.30%) had systemic lupus erythematous, two (2.30%) had nerve damage that had previously been diagnosed, one (1.15%) had rheumatoid arthritis, and one (1.15%) had connective tissue diseases. Eleven patients (12.64%) had previous total abdominal hysterectomy. The majority of our patients were primary POP; only 2% of patients had recurrent disease. Table 1 displays the demographic characteristics of the patients. In this retrospective chart review, 12 patients (13.79%) had stage I, 47 (54.02%) stage II, 24 (27.69%) stage III, and 4 (4.60%) stage IV prolapse. Individual compartment prolapse stages are shown in Table 2.

**Table 1.** Demographic characteristics of premenopausal women with pelvic organ prolapse.

	All patients (*n* = 87)
Age (years)	44.81 (4.9)
BMI (kg/m^2^)	24.48 (3.28)
Parity, *n* (%)	
Nulliparity	7 (8.05)
Primiparous	16 (18.4%)
Multiparous	64 (73.6%)
2 births	54 (62.1%)
3 births	6 (6.9%)
4 births	3 (3.4%)
5 births	1 (1.1%)
History of vaginal delivery, *n* (%)	
yes	79 (90.80)
no	8 (9.20)
Underlying disease, *n* (%)	
No	65 (74.71)
Diabetes millitus	8 (9.20)
dyslipidemia	5 (5.75)
thyroid disease	3 (3.45)
SLE	2 (2.30)
Rheumatoid arthritis	1 (1.15)
Recognized nerve damage	2 (2.30)
Connective tissue disease	1 (1.15)
Previous hysterectomy, *n* (%)	
yes	11 (12.64)
no	76 (87.36)
Previous pelvic floor repair, *n* (%)	
yes	2 (2.30)
no	85 (97.70)

**Table 2.** Degree and number of compartments of pelvic organ prolapse.

	All patients (*n* = 87)
Stage of pelvic organ prolapse	
I	12 (13.79)
II	47 (54.02)
III	24 (27.69)
IV	4 (4.60)
Anterior compartment prolapse	
I	22 (25.29)
II	42 (48.28)
III	21 (24.14)
IV	2 (2.30)
Postrior compartment prolapse	
I	52 (59.77)
II	29 (33.33)
III	5 (5.75)
IV	1 (1.15)
Apical compartment prolapse	
I	64 (73.56)
II	8 (9.20)
III	11 (12.64)
IV	4 (4.60)
Genital hiatus (cm)	3.93 (0.93)
Perineal body (cm)	2.71 (0.64)
Total vaginal length (cm)	7.21 (0.75)
Brink scores	8.16 (2.58)
Number of compartment prolapse	
1	46 (52.87)
2	32 (36.78)
3	9 (10.34)

*POP stage and compartment were determined using the standardized POP-Q system by trained urogynecologists*.

Analysis of symptom-specific bother scores based on the PFBQ (Table 3) revealed that vaginal bulging (item 6) was the most commonly reported symptom, present in 98.9% of patients, with more than 65% rating it as moderately to severely bothersome. Urinary frequency (item 2) and urgency (item 3) were also frequently experienced (82.8 and 65.5%, respectively), with over 40% of affected patients describing them as at least moderately bothersome. Although stress urinary incontinence (item 1) was reported by 67.8% of patients, the majority (44.8%) considered it only slightly bothersome. These findings suggest that prolapse-related and overactive bladder symptoms were more distressing than incontinence or bowel symptoms in this cohort. Full results for each PFBQ domain are presented in Table 3.

**Table 3.** The scores from a global pelvic floor symptom bother questionnaire.

	Not experienced symptoms (answer: no)	Experienced symptoms (answer: yes)				
		not bother at all	only a little bit	somewhat	moderate amount	a lot bother
PFBQ item1	28 (32.18)	10 (11.49)	29 (33.33)	9 (10.34)	7 (8.05)	4 (4.60)
PFBQ item2	15 (17.24)	6 (6.90)	15 (17.24)	15 (17.24)	20 (22.99)	16 (18.39)
PFBQ item3	30 (34.48)	2 (2.30)	12 (13.79)	12 (13.79)	18 (20.69)	13 (14.94)
PFBQ item4	29 (33.33)	10 (11.49)	12 (13.79)	9 (10.34)	18 (20.69)	9 (10.34)
PFBQ item5	40 (45.98)	6 (6.90)	9 (10.34)	12 (13.79)	9 (10.34)	11 (12.64)
PFBQ item6	1 (1.15)	4 (4.60)	9 (10.34)	16 (18.39)	33 (37.93)	24 (27.59)
PFBQ item7	48 (55.17)	4 (4.60)	9 (10.34)	7 (8.05)	12 (13.79)	7 (8.05)
PFBQ item8	59 (67.82)	0 (0)	14 (16.09)	6 (6.90)	5 (5.75)	3 (3.45)
PFBQ item9*	33 (37.93)*	26 (29.89)	10 (11.49)	8 (9.20)	3 (3.45)	7 (8.05)

PFBQ item9*: sexually inactive.

**Figure 1.**
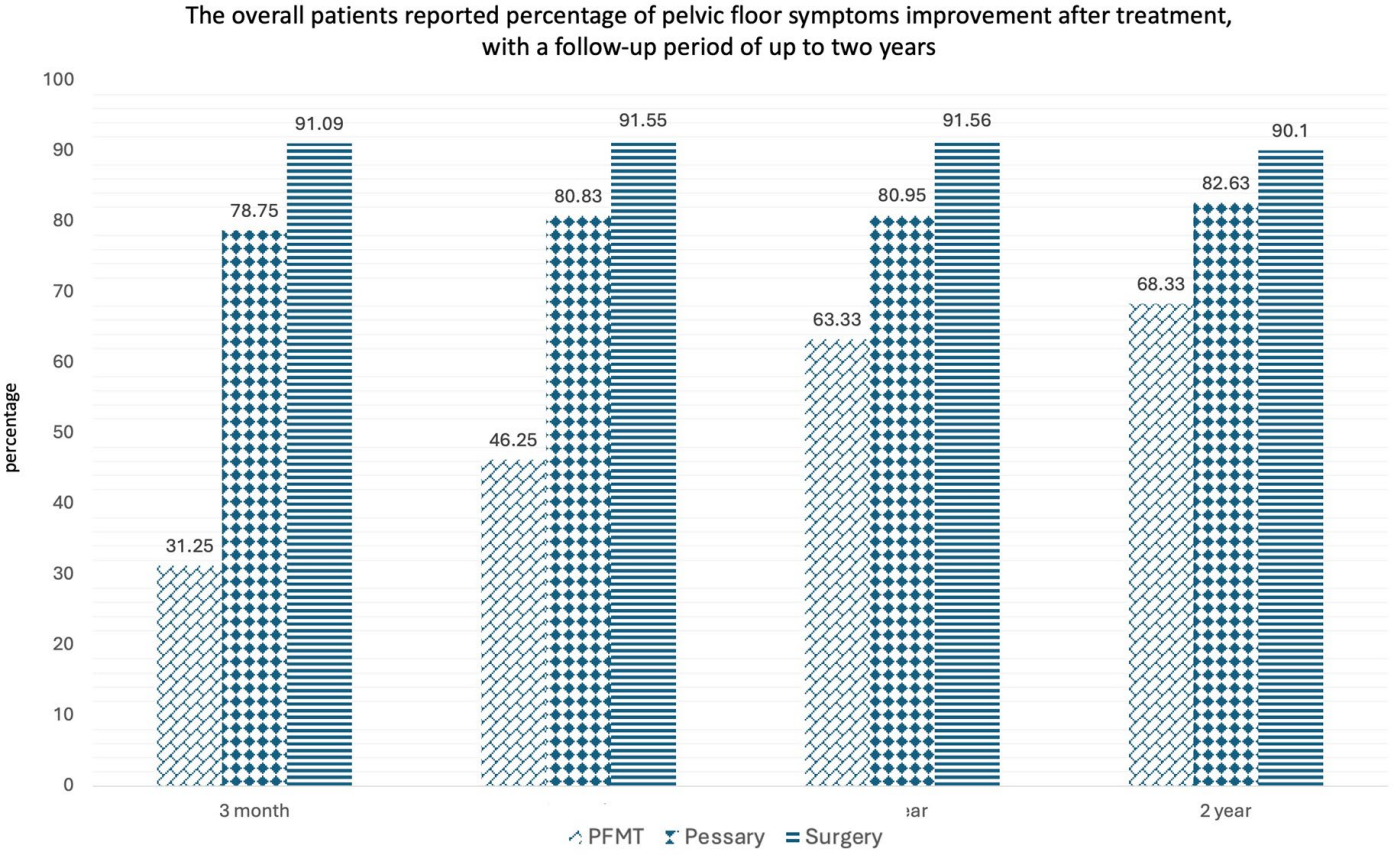
The overall patients reported percentage of pelvic floor symptoms improvement after treatment, with a follow-up period of up to two years.

All premenopausal women with POP were counseled on the choice of treatment by the healthcare provider team, consisting of urogynecologists, fellows, and specialty nurses, with shared decision-making with the patient, couple, and relatives. In this study, 9 patients (10.34%) chose pelvic floor muscle exercise and follow-up clinical symptoms; 24 patients (27.59%) used pessary devices; and the remaining 54 patients (62%) received surgical treatment. Vaginal hysterectomy with pelvic floor repair with uterosacral ligament plication (22 patients) is the most common procedure, followed by vaginal hysterectomy with pelvic floor repair with sacrospinous ligament fixation (13 patients). Interestingly, 8 patients decided to receive vaginal hysterectomy and total colpocleisis. (Table 4) Among the 9 women who selected pelvic floor muscle training (PFMT) as initial treatment, 2 switched to surgical intervention within 1 year, and 2 more by the 2-year follow-up. Of the 24 women who initially chose pessary management, one discontinued use by 3 months, three more by 1 year, and another three by 2 years, totaling seven discontinuations (29.2%) by the end of follow-up. From the retrospective cohort, the patient-reported percentage of improvement in pelvic floor symptoms following treatment with Pelvic Floor Muscle Training (PFMT), Pessary, or Surgery, across follow-up periods of 3 months, 1 year, and 2 years. Follow-up was routinely scheduled at 3, 12, and 24 months post-treatment. All 87 patients had complete data at these key timepoints, and no loss to follow-up was observed. Surgery achieved the highest reported improvement rate (91.09%) at the 3-month follow-up, and pessary (78.75%) and PFMT (31.25%). This early difference likely reflects the more immediate structural correction achieved with surgery, whereas pessary and PFMT may require longer durations to achieve their full effect. The efficacy of Surgery remained high at 91.55% at the 1-year follow-up, while Pessary and PFMT demonstrated modest improvements, with reported improvement rates of 80.83 and 46.25%, respectively. Surgery maintained its dominant position with an efficacy of 90.1% at the 2-year follow-up, followed by Pessary at 82.63% and PFMT at 68.33%. No recurrence of prolapse was observed among women who underwent surgical treatment during the 2-year follow-up period. These percentages reflect patients who continued with the respective treatments at each time point. (Figure 1).

**Table 4.** Treatment of pelvic organ prolapse in premenopausal women.

Treatment type	*n* (%)
Pessary	24 (28)
Pelvic floor muscle exercise	8 (10)
Surgery	55 (63)
Manchester operation	1 (1.8)
Laparoscopic sacrocervicopexy	2 (3.6)
Laparoscopic sacrohysteropexy	4 (7.3)
Laparoscopic USLS	3 (5.5)
Sacrospinous hysteropexy	2 (3.6)
VH and total colpocleisis	8 (14.5)
VH with SSLF	13 (23.6)
VH with US plication	22 (40)

*VH: Vaginal Hysterectomy, USLS: Uterosacral Ligament Suspension, SSLF: Sacrospinous ligament fixation*.

## Discussion

The results of our study shed light on the significant effects linked to POP, which comprise lower urinary tract symptoms (LUTS), bowel symptoms, and sexual disturbances among young women in Thailand. This is one of the largest studies investigating all treatments received among premenopausal patients with POP. The development of POP is influenced by numerous factors, such as advanced age, parity, and prolonged increased abdominal pressure from some personal factors such as occupation, obesity, or chronic cough [3,14]. Although our research was limited to premenopausal women, the mean age of the participants was 44 years old, and the majority had experienced symptoms for a duration of 1–4 years. Our research had an increase in patients’ ages compared with previous reports [5]. A subset of our patients exhibited pre-existing risk factors for the development of POP, given that 80% of them were multiparous women and had a mean body mass index of 24 kg/m^2^. It has been demonstrated that multifactorial factors such as increasing parity and overweight significantly influence the pathophysiology of POP. In the previous study in the Congo, they reported a mean age of 34 years, and most of the population had a BMI lower than 20, which was significantly lower than our study [5]. One reason that could lead to that study was malnutrition, which also plays a particular role in leading to POP in some populations [6]. Moreover, a challenge for healthcare for premenopausal women with POP is the disease associated with POP in young women, such as connective tissue disease. In this study, some of the patients had underlying diseases that led to factors that developed POP in the young, including rheumatoid arthritis, connective tissue diseases, and damaged nerves. These comorbidities may decrease collagen concentration, suggesting a different organization of the endopelvic connective tissue extracellular matrix, which increases the risk of developing POP at a younger age [15,16].

In this study, we have the number of premenopausal women with POP 87 cases during a 6-year period. During this period, the number of new cases of POP was 1567. Although premenopausal women accounted for 5.6% of POP cases treated at our center, this hospital-based proportion does not represent the population-level prevalence, which requires broader epidemiological studies. The reports on the prevalence of premenopausal POP are still limited due to the number of literatures. There is a literature reported as a case series of 24 cases in 4 years [17], and the highest number was reported in the Democratic Republic of the Congo with 107 patients in a prospective study during a 2-year period [5]; however, they were included only POP patients who came to receive surgical management, which did not represent the real prevalence in that study. Pelvic floor symptoms among women with POP are a wide range of symptoms associated with lower urinary tract symptoms (LUTS), bowel symptoms, and sexual dysfunction. All women with POP experience symptoms that vary in severity and impact their quality of life. Premenopausal women or young women may have unique pelvic floor symptoms and disturbances due to their age, work, and lifestyle. The study emphasizes the pelvic floor symptoms among premenopausal women, with vaginal bulging sensation being reported by 86 out of 87 patients. Furthermore, over 50% of the patients indicated that these symptoms were at least moderately bothersome. This is consistent with the findings of Gyhagen et al. who reported that bulging sensation is a significant symptom in younger age groups compared to the elderly in POP patients [8], noticed that despite increasing anatomical POP, patients are more likely to experience higher symptom burden that may plateau or decrease at an age corresponding to menopause [18,19]. Furthermore, several studies have demonstrated that the majority of patients experience vaginal bulging, which negatively impacts their quality of life, beginning when the leading point of prolapse is −1 or higher than the hymenal ring. According to a study of women with POP who reported accessing care, many women reported that health care providers were uncaring or unappreciative of the impact of their condition [20]. One possible explanation for this is that women did not comprehend the repercussions of their POP, particularly considering their age and the general absence of overt vaginal atrophy. This observation aligns with prior reports indicating that younger women with POP may experience stigma, misattribution, or lack of awareness [21,22]. A quarter of patients reported stress urinary incontinence and two-thirds reported overactive bladder symptoms as lower urinary tract symptoms (LUTS), with nearly half of these patients perceiving the symptoms as “somewhat” bothersome. It is not uncommon for LUTS and POP to coexist; however, premenopausal women with POP require comprehensive care and attention to the long-term consequences [23]. For example, in patients with SUI, the percentage of improvement of SUI following repair POP, in addition to the complications and long-term outcomes of concomitant anti-incontinent procedures, should be carefully advised for patients’ decision, particularly in this era of increasing innovations and evidence regarding the treatment of LUTS in younger age. These results suggest that in premenopausal women, surgical management provides not only rapid but also sustained symptom relief over time. This may explain why surgery is often favored by younger patients, who may perceive it as a definitive and long-term solution. In contrast, conservative approaches such as pelvic floor muscle training (PFMT) may require more time to yield meaningful improvement, while pessary use offers a relatively stable but intermediate level of benefit. However, pessary use may be less acceptable among sexually active young women due to concerns related to comfort and sexual function. This trend likely reflects age-specific differences in treatment goals, daily activity demands, and expectations around body image, intimacy, and fertility. Many of the women in this cohort were still working, managing family responsibilities, or considering future pregnancies. Therefore, individualized treatment counseling is especially important in this group, where the balance between efficacy, invasiveness, recovery time, and lifestyle impact must be carefully considered. In addition to sexual problems, pelvic organ prolapse has been linked to sexual dysfunction, including reduced libido, decreased frequency of sexual encounters, and difficulty achieving orgasms [24,25]. Several studies have linked menopause and POP to an increased prevalence of sexual dysfunction among women [26,27]. Almost forty percent of the premenopausal female participants in this study reported being sexually inactive. It is worth mentioning that POP may constitute a significant factor that disrupts their sexual lives, potentially through body image concerns, dyspareunia, or fear of worsening symptoms. This is consistent with previous studies that have reported negative impacts of POP on sexual function, even among younger women [7,28]. Pelvic floor dysfunction can result in symptoms such as discomfort in vagina, urinary or bowel loss, and the possibility of the pelvic organs descending or exiting through the vagina. The aforementioned elements have the potential to impact a woman’s sexual function by causing discomfort, disappointment, or even rejection from her companion or from herself. To our knowledge, while previous studies such as Durnea et al. [29] have included larger samples of premenopausal women, our study represents one of the largest cohorts specifically focusing on symptomatic premenopausal women with clinically diagnosed POP who required treatment. Furthermore, it is among the few studies from Southeast Asia to describe detailed pelvic floor symptoms and real-world treatment outcomes in this population. In addition, this study evaluates the effects of POP on a broad spectrum of pelvic floor symptoms and all types of treatment, including conservative treatment, pessary, and surgical management, which no prior research has addressed. Although PFBQ was used to assess baseline symptom burden in all participants, domain-specific follow-up data were not consistently documented due to the retrospective nature of this study. In our routine clinical practice, follow-up assessments typically emphasize global symptom improvement, which is directly recorded in the electronic medical record. While this represents a limitation in terms of symptom-level granularity, the use of global outcomes offers a practical and clinically intuitive metric for evaluating overall treatment effectiveness. This approach may also enhance applicability in real-world settings, where decision-making often relies on patient-perceived improvement rather than structured symptom scoring. As such, only global improvement rates were available and analyzed at post-treatment time points. This study was retrospective and descriptive in nature, and the sample size was limited by the total number of eligible cases available during the six-year study period. No a priori sample size calculation was performed. While this approach allowed us to include the complete cohort of premenopausal women with POP at our institution, it also limited the ability to conduct inferential analyses with sufficient statistical power. Nevertheless, this research has a number of limitations. To begin with, the data were gathered retrospectively from medical records. Sexual dysfunction and other pelvic floor symptoms could potentially be improved through the use of questionnaires such as the female sexual function index. Due to the descriptive and exploratory nature of this study in a relatively uncommon population, multivariate modeling to control for confounding factors was not performed. The limited sample size, along with variable completeness of domain-specific follow-up data, constrained the ability to conduct robust regression analyses. Future prospective studies with larger sample sizes and comprehensive follow-up should explore the independent effects of clinical variables on treatment outcomes in premenopausal women with POP. Moreover, the authors intend to conduct additional qualitative research on pelvic floor symptoms in premenopausal women who have experienced POP.

## Conclusion

In general, this study emphasizes the profound impact that symptoms of the pelvic floor have on the reproductive health and quality of life of premenopausal women in the Asian population. The characteristics and pelvic floor bother symptoms of Thai women with POP were reported. The severity of POP and its negative effects on pelvic symptoms are highlighted.

## Data Availability

The data that support the findings of this study are available from the corresponding author upon reasonable request. The data are not publicly available due to privacy or ethical restrictions. The datasets used and analyzed during the current study available from the corresponding author on reasonable request to orawee.chi@mahidol.ac.th.
